# Gynecologic surgery for benign disease: Preserving reproductive potential

**DOI:** 10.1002/ijgo.70546

**Published:** 2025-09-24

**Authors:** Togas Tulandi, Edgar Mocanu, Nikhil Purandare, Scott M. Nelson, Eytan R. Barnea, Ruth Dolmo Carluccio, Elif Goknur Topcu, Dov Feldberg, FIGO Committee on Reproductive Endocrinology and Infertility, Nikhil Purandare, M. Louise Hull, Ajey Bhardwaj, Andres Calle, Dov Feldberg, Edgar Mocanu, Eytan Barnea, Goknur Topcu, Hrishikesh Pai, Henry Laurie, Ivonne Jeannette Diaz Yamal, Jaideep Malhotra, Gitau Mburu, Scott Nelson, Togas Tulandi

**Affiliations:** ^1^ Department of Obstetrics and Gynecology McGill University Montreal Quebec Canada; ^2^ Rotunda Hospital Dublin Ireland; ^3^ Royal College of Surgeons in Ireland Dublin Ireland; ^4^ Department of Obstetrics and Gynaecology Galway University Hospital Galway Ireland; ^5^ School of Medicine, Dentistry and Nursing University of Glasgow Glasgow UK; ^6^ University of Miami Miller School of Medicine New York New York USA; ^7^ Department of Obstetrics, Gynecology and Reproductive Sciences University of Miami Miller School of Medicine Miami Florida USA; ^8^ Sisli Kolan International Hospital Istanbul Turkey; ^9^ Helen Schneider Hospital for Women Rabin Medical Center Petah Tikva Israel; ^10^ Tel Aviv University School of Medicine Tel Aviv Israel

**Keywords:** benign gynecologic surgery, fertility‐sparing surgery, FIGO, infertility, reproductive function, reproductive surgery

## Abstract

Preserving fertility is essential when managing benign gynecologic disorders in reproductive‐aged women. Surgical interventions can impact future fertility, therefore requiring an evidence‐based, individualized approach. The FIGO Committee on Reproductive Endocrinology and Infertility reviewed current literature to develop recommendations for fertility‐sparing surgical management. Effective treatment must balance disease control with fertility preservation. Evidence supports refined surgical techniques and alternative methods that minimize reproductive harm. In pregnancy loss management, medical treatment is preferred; however, if surgery is needed, hysteroscopic evacuation is safer than sharp curettage, reducing the risk of intrauterine adhesions. Similarly for other operative hysteroscopic procedures or abdominal procedures, including myomectomy, the use of an adhesion‐reducing substance is recommended. During abdominal myomectomy, only symptomatic or fertility‐impairing fibroids should be removed, as excessive resection may reduce pregnancy chances. In endometrioma surgery, preserving ovarian tissue is crucial by minimizing stripping of the pseudocapsule, or using sclerotherapy may help. Oocyte cryopreservation should be discussed when recurrence or reduced ovarian reserve is a concern. Hydrosalpinx often requires salpingectomy to enhance in vitro fertilization outcomes, but surgeons should protect ovarian blood flow by minimizing thermal injury. For adenomyosis, medical management is preferred due to the risks associated with surgical techniques like the triple‐flap procedure. In polycystic ovary syndrome, laparoscopic ovarian drilling is discouraged; ovulation induction with letrozole or gonadotropins is safer and effective. In summary, fertility‐sparing treatment demands personalized, evidence‐based strategies that prioritize reproductive potential while managing disease. As reproductive goals evolve, continued refinement of these approaches remains vital to women's health care.

## 1 INTRODUCTION

Gynecologic disorders commonly affect women of reproductive age, with conditions such as uterine fibroids occurring in about 50% of reproductive‐aged women, ovarian endometriomas in 17%–44% of women with endometriosis, and polycystic ovary syndrome (PCOS) affecting about 10% of reproductive‐aged women.^1^ While surgical intervention may be necessary to address symptoms or treat disease, the primary objective in reproductive‐aged women must be to remedy the gynecologic condition while preserving fertility potential. This dual imperative is particularly critical for women with existing infertility or those planning future conception.

Contemporary gynecologic surgery increasingly utilizes minimally invasive approaches, which generally offer advantages in terms of recovery time, adhesion formation, and reproductive outcomes compared with open procedures. However, even minimally invasive techniques can compromise fertility if not performed with fertility‐preservation principles. Understanding the anatomy and physiology of the reproductive tract is fundamental, and any surgery performed in women of reproductive age must ensure that fertility is not jeopardized. Adequate surgical training, evidence‐based techniques, and systematic audit of reproductive outcomes are essential components of quality care.

The purpose of this paper is to highlight the importance of reproductive function‐sparing gynecologic surgery for common benign conditions and provide evidence‐based recommendations for achieving optimal outcomes (Table 1). This article focuses on minimally invasive surgical approaches, as these represent current standard practice for most gynecologic conditions in reproductive‐aged women.

**TABLE 1:** Recommendations for reproductive function‐conserving gynecologic surgery for common benign conditions.

Procedures	Recommendation
Dilation and curettage (D&C)	Vacuum curettage is better than sharp curettage. Instead of D&C for removal of retained products of conception, hysteroscopic removal is recommended
LEEP and cone biopsy	Minimize the depth of cone biopsy to <10 mm if feasible
Operative hysteroscopy	After hysteroscopic myomectomy for >1 myomas apposing each other, insert an intrauterine balloon followed by a high dose of estrogen vaginally for 3 weeks. Use of intrauterine adhesion‐reducing substances has been advocated
Abdominal myomectomy	For multiple myoma, not all myomas should be removed. Use adhesion‐reducing substances
Excision of endometrioma	Pseudocapsule adherent to the ovarian tissue should not be stripped. Use minimal cautery and opt for suturing for hemostasis
Salpingectomy for hydrosalpinx	Use the least thermogenic instrument and perform dissection close to the fallopian tube
Adenomyosis	Triple‐flap method for diffuse adenomyosis is associated with considerable risk of uterine rupture. Medical treatment is preferable
Laparoscopic ovarian drilling	Should be used sparingly

## 2 DILATION AND CURETTAGE

Dilation and curettage (D&C) is commonly performed to manage pregnancy loss and remove retained products of conception or retained placental tissue. However, this procedure carries significant risk for intrauterine adhesions leading to reduced reproductive outcome.^2^ In a French multicenter study, the incidence of intrauterine adhesions after D&C using plastic suction aspirator was 18.4%.^3^ When comparing vacuum aspiration with hysteroscopic morcellation, intrauterine adhesion rates were similar, although vacuum aspiration was associated with higher retained tissue, requiring another procedure.^4^ Hysteroscopic removal of the products of conception is a superior procedure compared to D&C using a metal curette for preserving future fertility, including a lower risk of intrauterine adhesions.^5^ Indeed, the World Health Organization (WHO),^6^ as well as other organizations, recommends against performing sharp blind curettage in pregnancy‐related conditions. Repeated D&Cs may lead to increased intrauterine adhesions. In order to reduce intrauterine adhesion formation, the use of intrauterine adhesion‐preventing substances has been advocated.^3^ The FIGO–GCH joint consensus statement also discourages the use of blind intrauterine procedures in the evaluation and management of women with suspected intrauterine pathologies.^7^

### 2.1 Recommendation

1. Medical management should be the first‐line treatment for pregnancy loss.
2. When surgical intervention is necessary, hysteroscopic removal of retained products of conception is preferred over traditional D&C.
3. Sharp curettage should be avoided due to the increased risk of adhesions and inferior reproductive outcomes.

## 3 LOOP ELECTROSURGICAL EXCISION PROCEDURE AND CONE BIOPSY

Women of reproductive age with low‐risk early‐stage cervical cancer are eligible for fertility‐sparing treatment options such as loop electrosurgical excision procedure (LEEP) or cone biopsy when lesions are more extensive. These procedures are essential for preserving patient health, preventing cervical cancer progression, and enabling future conception. However, these procedures do not come without complications. A multicenter study showed that an interval of less than 12 months between LEEP and conception increases the risk of pregnancy loss.^8^ Bjørge et al.^9^ reported an increase in spontaneous pregnancy loss and preterm birth following both of these excisional procedures. Heinonen et al.^10^ evaluated the impact of LEEP on risk for preterm delivery and found an increased risk after a single LEEP procedure, with the highest risk for preterm delivery after repeat LEEP. Maina et al.^11^ noted that the larger the area of excision, the worse the obstetric outcome, including increased risk of preterm delivery, premature rupture of membranes, and labor dystocia. When LEEP does not achieve the desired goal, cone biopsy is the next step, where part or the entire cervix is removed.

The risks associated with cone biopsy on future conceptions are further magnified. They include cervical stenosis, reduced fertility, labor dystocia, cervical insufficiency, and premature delivery. These risks could be minimized by limiting the depth of biopsy to less than 10 mm, if feasible.^12^

Excisional procedures for the cervix (LEEP or cervical conization) are methods to treat cervical intraepithelial neoplasia (CIN) or high grade squamous intraepithelial lesions (HSIL). The incidence of cervical stenosis after conventional LEEP was 8% in one study^13^ and after cryotherapy or laser ablation it was less than 1%. It also depends on the depth of incision (over 10 mm) and the amount of tissue removed. Periodic cervical dilation to minimize cervical stenosis has been recommended.^14^

## 4 OPERATIVE HYSTEROSCOPY

Hysteroscopy is a valuable approach for diagnosing and treating uterine anomalies and other pathologies, including endometrial polyps, submucous myomas, intrauterine adhesions, and endometrial malignancies. Diagnosis and treatment of these pathologies can lead to spontaneous pregnancy and improve fertility outcomes, as well as improving patient health and quality of life. However, intrauterine instrumentation appears to be one of the major causes of intrauterine adhesion formation.

The main cause of intrauterine adhesions related to operative hysteroscopy is hysteroscopic resection of multiple myomas. In a study of 217 women who had undergone hysteroscopic myomectomy, the incidence of adhesion formation at second‐look hysteroscopy was 10.3% after resection of a solitary myoma and 36.4% after removal of more than one myoma in apposition to each other.^15^ For the latter, the use of an intrauterine balloon followed by a high dose of estrogen vaginally for 3 weeks and application of intrauterine adhesion‐reducing agents has been advocated.

### 4.1 Recommendations

1. Hysteroscopic surgery requires adequate preoperative assessment of pathology to reduce the risks of surgery, including cervical or uterine perforation and related complications, intrauterine adhesions, infection, and excessive bleeding requiring hysterectomy.
2. Some adhesion‐reducing products and temporary mechanical uterine wall separation may reduce the risk of intrauterine adhesion formation.
3. Adhesiolysis should be performed using cold scissors to reduce the risk of recurrent intrauterine adhesion formation.

## 5 MYOMECTOMY

The objective of myomectomy in reproductive‐aged women is to remove symptomatic myomas while preserving uterine integrity and fertility potential. Most women with uterine myomas are asymptomatic and do not require surgical intervention. However, myomectomy may be indicated for women with severe menorrhagia, pressure symptoms, or myomas that impair fertility. A critical surgical decision involves whether to remove only symptomatic or fertility‐affecting myomas versus performing numerous excisions when multiple myomas are present.

While the temptation may exist to remove all visible myomas during surgery, evidence demonstrates that extensive myoma removal significantly compromises fertility outcomes. Shue et al.^16^ evaluated 144 women following abdominal myomectomy and found that removal of six or more myomas was associated with substantially reduced reproductive success compared to removal of fewer than six myomas. Pregnancy rates were 70.8% versus 22.9%, respectively, with women in the extensive removal group more likely to require assisted reproductive technology (ART). The mechanism underlying this fertility impairment likely involves disruption of uterine blood supply, leading to compromised uterine function, increased intra‐abdominal adhesion formation, and potential endometrial ischemia with resultant amenorrhea.

Myoma enucleation is facilitated by preserving the pseudocapsule, which consists of compressed surrounding myometrial tissue (intracapsular myomectomy).^17^ Multilayered uterine closure is optimized by using barbed sutures, which—compared with conventional sutures—are associated with reduced blood loss and shorter operative times.^18^ Given concerns about the potential dissemination of occult malignancies, we advocate for confined (contained) morcellation techniques for the removal of solid specimens during minimally invasive surgery.^19^

Essential technical considerations for fertility preservation include selective myoma removal, targeting only symptomatic or fertility‐impairing myomas, multilayered closure of uterine defects to restore anatomical integrity, and application of adhesion‐reducing substances to minimize postoperative adhesion formation. The risks of surgical complications from extensive myomectomy outweigh the potential benefits, emphasizing the importance of a conservative surgical approach.

### 5.1 Recommendations

1. **Patient selection:** Limit myomectomy to women with symptomatic myomas or those with myomas impacting fertility.
2. **Surgical approach:** Remove only myomas causing symptoms or fertility impairment; avoid excision of numerous small myomas.
3. **Technical considerations:**
  ⚬ Avoid removal of countless myomas when possible, to optimize fertility outcomes.
  ⚬ Employ multilayered suturing for uterine defect closure.
  ⚬ Apply adhesion‐reducing substances at surgery completion.
4. **Counseling:** Inform patients that removal of numerous myomas significantly reduces pregnancy rates and may necessitate ART.

## 6 REMOVAL OF OVARIAN ENDOMETRIOMA

Ovarian endometrioma management in reproductive‐aged women requires careful balance between symptom relief and preservation of ovarian function. Surgical excision of ovarian endometrioma consistently reduces ovarian reserve, particularly anti‐Müllerian hormone (AMH) levels, which are predictive of in vitro fertilization (IVF) success rates.^20^, ^21^ This ovarian reserve compromise has prompted investigation of fertility‐sparing alternatives to traditional cystectomy.

Alternative approaches to complete surgical excision include simple aspiration, aspiration with ablation, and sclerotherapy. While these techniques preserve more ovarian tissue than cystectomy, they are associated with higher recurrence rates. Sclerotherapy represents a promising middle‐ground approach, demonstrating AMH reduction that is less severe than complete cystectomy, while providing better long‐term control than aspiration alone.^22^ However, long‐term fertility outcomes with these conservative approaches require further investigation.

When surgical excision is necessary, fertility‐preserving techniques should be employed. The surgical approach begins with gentle separation of the pseudocapsule from healthy ovarian tissue. When the pseudocapsule is densely adherent to ovarian stroma, stripping should be discontinued to preserve functional ovarian tissue. Minimal bipolar electrocautery may be applied to the remaining cyst wall, although excessive thermal energy should be avoided. Hemostasis achieved through suturing is preferable to thermal coagulation to minimize additional ovarian damage.

Given the dual impact of endometriosis and surgical intervention on fertility potential, preoperative fertility counseling is essential. Oocyte or embryo cryopreservation should be discussed and offered before surgery, particularly for women with bilateral endometriomas, recurrent disease, or those planning to delay childbearing.

### 6.1 Recommendations

1. **Preoperative counseling:** Discuss fertility preservation options including oocyte/embryo cryopreservation before endometrioma surgery.
2. **Treatment selection:** Consider conservative management (sclerotherapy, aspiration) for asymptomatic women prioritizing fertility preservation.
3. **Surgical technique when excision is required:**
  ⚬ Attempt gentle pseudocapsule separation initially.
  ⚬ Discontinue stripping when the capsule is densely adherent to preserve ovarian tissue.
  ⚬ Use minimal cautery and opt for suturing for hemostasis.
  ⚬ Avoid excessive thermal energy application.
4. **Patient counseling:** Inform patients of the inevitable reduction in ovarian reserve following surgical excision.
5. **Follow‐up:** Monitor AMH levels postoperatively and adjust fertility treatment plans accordingly.

## 7 SALPINGECTOMY FOR HYDROSALPINX

Hydrosalpinx significantly impairs IVF outcomes, with pregnancy rates reduced by approximately 50% compared to women without tubal pathology.^23^ The mechanism involves retrograde flow of hydrosalpinx fluid into the uterine cavity, which contains inflammatory mediators, bacterial endotoxins, and cytokines that compromise endometrial receptivity and embryo implantation. Several surgical approaches have been evaluated, including salpingostomy, proximal tubal occlusion, and salpingectomy.

Salpingostomy carries a high risk of tubal reocclusion and persistent fluid accumulation, while proximal tubal occlusion may lead to continued distension of the trapped tubal segment. Salpingectomy has emerged as the preferred approach, demonstrating superior pregnancy rates in subsequent IVF cycles, while providing the additional benefit of reducing ovarian cancer risk through elimination of potential fallopian tube origins.

However, salpingectomy poses potential risks to ovarian function through disruption of the tubo‐ovarian vascular anastomoses. Studies have demonstrated modest but significant reductions in ovarian reserve parameters following salpingectomy, particularly when extensive electrocautery is used.^24^ To minimize ovarian compromise, surgical technique should prioritize preservation of ovarian blood supply through judicious use of thermal energy and strategic instrument placement.

The optimal surgical approach involves identification of the plane between the fallopian tube and ovary within the mesosalpinx, with coagulation and division performed as close to the tubal serosa as possible. Minimal electrocautery should be used, with preference for sharp dissection where feasible. The utero‐ovarian ligament and medial aspects of the mesosalpinx should be preserved to maintain ovarian blood supply.^25^

### 7.1 Recommendations

1. **Indication assessment:** Salpingectomy is recommended for hydrosalpinx before IVF treatment to optimize pregnancy outcomes.
2. **Surgical technique:**
  ⚬ Perform dissection close to the fallopian tube to preserve ovarian blood supply.
  ⚬ Use minimal thermal energy and opt for sharp dissection when possible.
  ⚬ Preserve the utero‐ovarian ligament and medial mesosalpinx.
  ⚬ Avoid excessive electrocautery near the ovarian hilum.
3. **Patient counseling:** Inform patients of the modest risk of reduction in ovarian reserve and consider preoperative AMH assessment.

## 8 ADENOMYOSIS

Adenomyosis affects 20%–30% of reproductive‐aged women and is associated with significantly reduced fertility outcomes, including lower implantation rates, increased pregnancy loss rates, and reduced live birth rates in both natural conception and assisted reproduction.^26^ The condition may present as focal adenomyomas or diffuse disease, with treatment approaches varying accordingly.

Focal adenomyosis may be amenable to surgical resection using techniques similar to myomectomy, with careful attention to maintaining uterine wall integrity and avoiding excessive myometrium removal. However, diffuse adenomyosis presents greater therapeutic challenges, and surgical intervention carries substantial risks.

The triple‐flap technique for diffuse adenomyosis involves extensive myometrium resection, removing adenomyotic tissue and 1 cm margins of apparently normal myometrium, both superficially and deeply. While this approach may provide symptomatic relief, it is associated with significant complications, including uterine rupture in 5.7% of subsequent pregnancies, endometrial defects, and increased risk of placenta accreta spectrum disorders.^27^, ^28^, ^29^ The extensive nature of this procedure often results in a structurally compromised uterus with questionable functional capacity.

Contemporary management of adenomyosis in women desiring fertility has shifted toward medical therapy as the first‐line treatment. Pretreatment with gonadotropin‐releasing hormone agonists or antagonists for 3–6 months before assisted reproduction has demonstrated improved pregnancy outcomes with significantly lower morbidity than surgical intervention. This approach allows for disease suppression while preserving uterine integrity and function.

### 8.1 Recommendations

1. **Treatment hierarchy:** Medical management with gonadotropin‐releasing hormone agonists/antagonists should be the first‐line therapy for adenomyosis in reproductive‐aged women.
2. **Surgical considerations:**
  ⚬ Focal adenomyosis may be considered for surgical resection using fertility‐sparing techniques.
  ⚬ The triple‐flap technique for diffuse adenomyosis should be avoided due to a high risk of uterine rupture (5.7%).
  ⚬ Extensive myometrial resection compromises reproductive outcomes.
3. **Pre‐ART treatment:** Consider 3–6 months of hormonal suppression before IVF treatment.
4. **Patient counseling:** Discuss the significant risks of surgical intervention versus medical management options.

## 9 LAPAROSCOPIC OVARIAN DRILLING

Laparoscopic ovarian drilling (LOD) was historically performed for clomiphene‐resistant PCOS to reduce ovarian androgen production and restore ovulation. The procedure involves creating multiple punctures in the ovarian cortex using electrocautery or laser energy.^30^ While effective for inducing ovulation, accumulating evidence demonstrates significant detrimental effects on ovarian reserve and long‐term reproductive function.

Meta‐analyses consistently show that LOD results in substantial reductions in anti‐Müllerian hormone levels and antral follicle counts, indicating permanent ovarian reserve depletion.^31^ Additionally, the procedure is associated with periovarian adhesion formation, which may impair oocyte retrieval during assisted reproduction and potentially affect natural fertility through compromised ovum pickup.

Contemporary medical ovulation induction has largely superseded the need for LOD. Letrozole has emerged as first‐line therapy for ovulation induction in PCOS, demonstrating superior pregnancy and live birth rates compared to clomiphene citrate. Systematic reviews confirm that letrozole achieves equivalent live birth rates to LOD in clomiphene‐resistant PCOS, without the associated ovarian reserve compromise.^32^

The long‐term reproductive consequences of LOD remain uncertain, particularly regarding fertility after the first successful pregnancy. Given the availability of effective medical alternatives and the irreversible nature of ovarian damage from drilling, the procedure is no longer recommended in contemporary practice.

### 9.1 Recommendations

1. **Avoid routine use:** LOD should not be performed given the available medical alternatives.
2. **Medical alternatives:** Letrozole is the preferred first‐line therapy for ovulation induction in PCOS.
3. **Reserve considerations:** LOD causes permanent ovarian reserve depletion and periovarian adhesion formation.
4. **Treatment progression:** Utilize letrozole followed by gonadotropins and IVF as needed.

## 10 REPRODUCTIVE SURGERY TRAINING

The increasing prevalence of infertility and expansion of ART has paradoxically led to decreased emphasis on reproductive surgery training. However, surgical correction of anatomical factors remains fundamental to optimizing fertility outcomes and can significantly enhance the success of subsequent assisted reproduction.

Ideally, reproductive surgery should be performed by specialists trained in reproductive endocrinology and infertility (REI) with dedicated surgical expertise. In North America, reproductive surgery training is typically integrated within REI fellowships, with the newer minimally invasive reproductive surgery (MIRS) fellowship providing specialized focus on surgical techniques. The Royal College of Obstetricians and Gynaecologists offers Accredited Subspecialty Training in Reproductive Medicine and Surgery in the UK.

However, many reproductive‐aged women without diagnosed infertility undergo gynecological surgery performed by general gynecologic surgeons. It is crucial that all surgeons operating on reproductive‐aged women understand fertility‐preserving principles and customize procedures to maintain or enhance reproductive potential. The surgeon must balance therapeutic objectives with preservation of reproductive function, recognizing that more extensive procedures can be considered after family completion.

Continuing education in reproductive surgery principles is essential for all practitioners treating reproductive‐aged women. Professional societies including the American Society of Reproductive Medicine (ASRM), American Association of Gynecologic Laparoscopists (AAGL), European Society of Human Reproduction and Embryology (ESHRE), European Society of Gastrointestinal Endoscopy (ESGE), and the International Federation of Fertility Societies (IFFS) provide educational courses and hands‐on workshops to disseminate evidence‐based fertility‐sparing surgical techniques.

### 10.1 Recommendations

1. **Specialized training:** Reproductive surgery should ideally be performed by REI specialists with surgical training.
2. **General gynecology:** All gynecologic surgeons should understand fertility‐preserving principles.
3. **Continuing education:** Regular participation in reproductive surgery courses and workshops is essential.
4. **Quality assurance:** Systematic audit of reproductive outcomes following surgery should be implemented.

## 11 CONCLUSIONS

The management of gynecological disorders in reproductive‐aged women requires a fundamental paradigm shift from disease‐focused to fertility‐preserving approaches. The primary objective must be symptom relief and disease treatment while optimizing reproductive potential, recognizing that more extensive interventions can be deferred until after family completion.

Medical management should be considered the first‐line therapy for most benign gynecologic conditions affecting reproductive‐aged women. When surgical intervention is necessary, evidence‐based fertility‐sparing techniques must be employed, with careful patient selection and thorough preoperative counseling regarding reproductive implications.

Key principles include selective surgical intervention targeting only symptomatic or fertility‐impairing pathology, utilization of minimally invasive approaches when appropriate, application of fertility‐preserving surgical techniques, and consideration of fertility preservation options including gamete or embryo cryopreservation when ovarian reserve compromise is anticipated (Table 1).

The evolution of reproductive surgery requires ongoing education, systematic outcome auditing, and multidisciplinary collaboration between reproductive endocrinologists, gynecologic surgeons, and fertility specialists. Only through adherence to evidence‐based fertility‐sparing principles can we optimize both therapeutic outcomes and reproductive potential for women of reproductive age.

## AUTHOR CONTRIBUTIONS

TT, EM, and NP contributed to conceptualization, writing, editing, and finalizing the manuscript. SMN, ERB, RDC, and EGT contributed to writing, editing, and finalizing the manuscript. DF contributed to editing and finalizing the manuscript.

## CONFLICT OF INTEREST STATEMENT

The authors declare no conflicts of interest in relation to the article.

## FUNDING INFORMATION

No funding received.

## Data Availability

Data sharing is not applicable to this article as no new data were created or analyzed in this study.
